# An Inexpensive model to teach hemorrhage control in resource limited settings

**DOI:** 10.12669/pjms.37.3.3517

**Published:** 2021

**Authors:** Omar Abbas Ahmed Malik, Rijah Chhapra

**Affiliations:** 1 Dr. Omar Abbas Ahmed Malik, MBBS, Patients’ Aid Foundation, Jinnah Postgraduate Medical Center, Karachi, Pakistan; 2 Dr. Rijah Chhapra, MBBS, Patients’ Aid Foundation, Jinnah Postgraduate Medical Center, Karachi, Pakistan

**Keywords:** Hemorrhage, Manikin, Exsanguination, Trauma

## Abstract

We have created a bleeding leg simulator using inexpensive and readily available materials to teach civilians in resource-poor settings how to control exsanguinating hemorrhage until the patient can be brought to the hospital, as commercially available mannequins are often too expensive in these settings. Items used include a leg of lamb, IV tubing, IV fluids, and food coloring.

The model was consistently rated as ‘‘nearly – real’’ to ‘‘life like’’ by ten physicians and surgeons, cost less than fifty dollars to make, and provided a fairly realistic model for teaching hemorrhage control.

## INTRODUCTION

Trauma is one of the leading causes of death worldwide, with exsanguinating hemorrhage possibly the most likely preventable cause^1^-^3^ especially in lower- and middle-income countries (LMICS) where Emergency Medical Services systems are underdeveloped or absent. This is certainly a problem in Pakistan, where countless limbs and lives might be saved if appropriate measures were taken in a timely manner to stop severe bleeding from various injuries until the patient can be transported to the hospital.

### Importance:

Teaching civilians how to control exsanguinating hemorrhage until the patient can be brought to the hospital could save innumerable lives, either as stand-alone classes or as a part of courses like Until Help Arrives (promoted by the American College of Emergency Physicians).

Unfortunately, mannequins and their replacement parts can be too expensive for institutes or organization in LMICS.

### Goals:

To describe a simple, low – cost alternative to commercially available mannequins to teach hemorrhage control using simulated scenarios so that they are confident enough to utilize these skills should the need arise.

## INNOVATION

Below, we describe an easy method to make a simple bleeding simulator, to teach hemorrhage control, using inexpensive and readily available items:

1. 1 x goat / lamb hind leg
2. Cling film
3. Tunneler from a tunneled dialysis catheter set
4. 2 x IV tubing
5. 2 x bottles / bags of saline or other fluid
6. Red food coloring
7. 1 x 5cc syringe
8. A knife
9. Some shirt / tights / pants to cover the leg (if desired)

**Fig.1:**
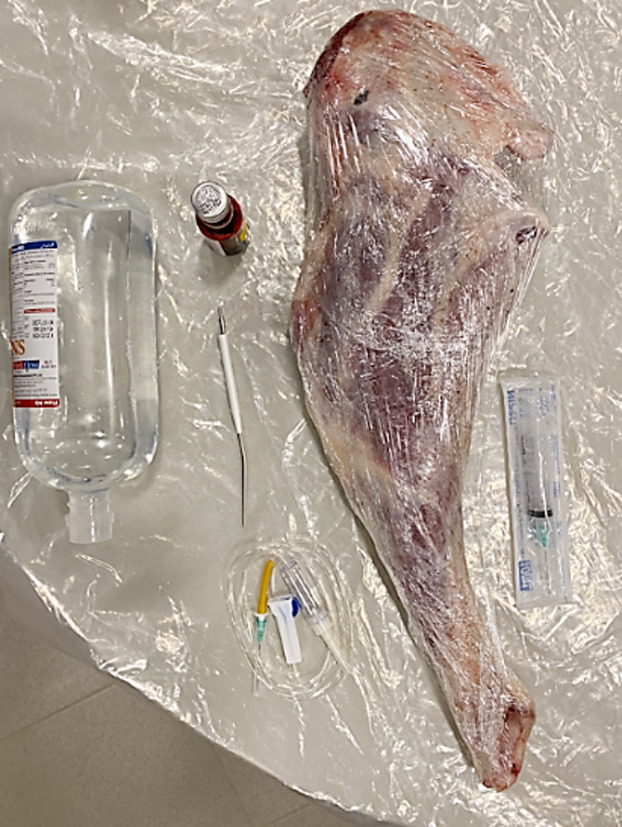
Goat leg wrapped in cling film, IV tubing, Tunneler, Saline, Red food coloring, syringe.

The process requires one to simply make a few incisions in the meat leg and pass IV tubing (connected to bags of food coloring) through them to act as vessels and cause the simulated blood to flow out of the wounds. The steps are detailed below:

### Steps:

**Fig.2:**
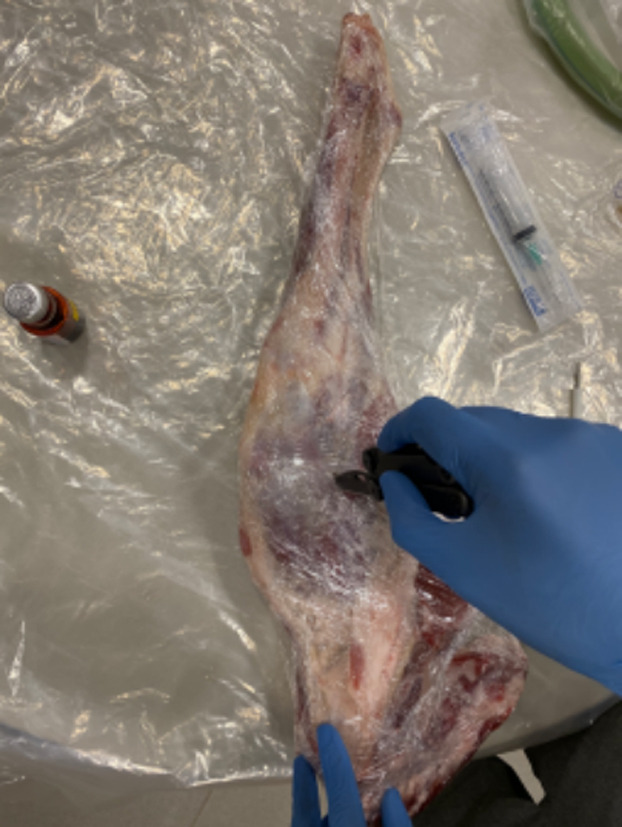
Creating the “wounds”.

First, partially defrost the leg of lamb and wrap it in cling film / saran wrap. Then, use the knife to make a simple stab wound on one side, and a deeper / larger wound on the other side (Fig.2).

Now, take the plastic connecter off of the IV tubing and connect it to the tunneler (Fig.4).

**Fig.3:**
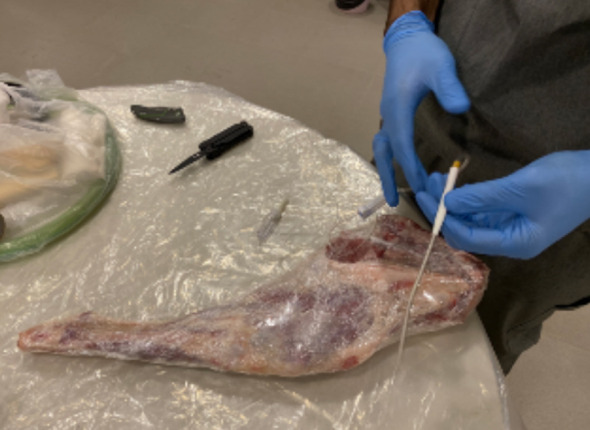
Connecting the IV tubing to the tunneler.

**Fig.4:**
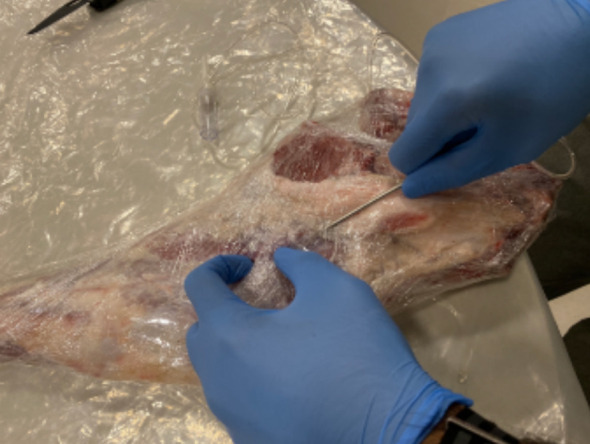
Using the tunneler to place the IV tubing into the wound.

Use the tunneler to puncture the meat a few inches proximal to the ‘‘wound’’ and create a tunnel to the wound, pulling the IV tubing into this tunnel (Fig.3).

Once you have pulled the tunneler out of the wound, detach the tubing and retract it slightly so that it is hidden in the tunnel. Repeat this procedure on the other side for the other wound. Attach the IV tubing to the fluid which has been mixed with red food coloring or other similar dye.

**Fig.5:**
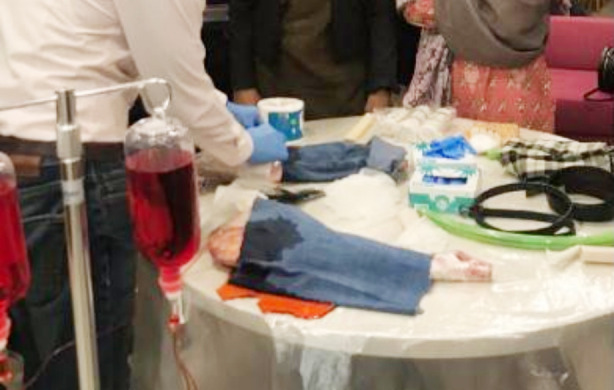
Model in use.

At this point, you may choose to put some sort clothing on the leg if you so desire. Finally, you can control the rate and amount of bleeding with the roller – clamp on the IV tubing, and even simulate arterial bleeding by manually compressing the bag in a pulsatile manner (Fig.5).

The mannequin was shown to 5 attending – level surgeons and 5 EM residents to assess realism before using it to teach civilians.

The model was consistently rated as ‘‘nearly – real’’ to ‘‘life like’’ by all physicians and surgeons on a rating scale of 1-5 that assessed: realistic bleeding, ‘tactile’/feel, response to intervention, and applicability to real-life scenarios. 1 was unrealistic and 5 was considered life-like.

This model cost us less than 50 dollars to make, compared to commercial models that can cost upwards of several hundred dollars.

## DISCUSSION

Our literature review was unable to find an improvised simulator that worked like ours, and could be used for teaching multiple hemorrhage control skills, though we were able to find one paper describing a hybrid model for control of groin hemorrhage,^4^ and another one (that seemed less realistic) for teaching tourniquet application.^5^

We used this model in three training sessions with civilians, and one with new nurses and Operating Room technicians. The majority of participants felt confident in their improved ability to control life-threatening hemorrhage both in and out of the hospital based on the “Until Help Arrives” Post-Course Survey [supplement attached].It has been shown that similar training methods are effective in teaching such life-saving skills, and may improve chances of survival for many patients that would not otherwise have immediate access to emergency care.^6^

### Limitations of the study:

It must be recognized that our method for assessing the effectiveness of this course was semi-quantitative and has not yet been shown to improve real-world outcomes. Further, the number of medically trained assessors was limited.

## CONCLUSIONS

Despite these limitations, we believe that this model puts such training sessions within reach of many centers and communities in LMICS as it is fairly inexpensive and can be used to teach a lifesaving skill in settings where it is direly needed and equally difficult to afford to do so.
